# Incidental Discovery of a Chronically Ruptured Ovarian Dermoid in a Patient With Right Upper Quadrant Pain

**DOI:** 10.7759/cureus.26035

**Published:** 2022-06-17

**Authors:** Mussanna Ahmed, Mossum Sawhney, Latika Baranga, Nasser Ali, Robert Sullivan

**Affiliations:** 1 Department of Radiology, State University of New York (SUNY) Downstate Health Sciences University, Brooklyn, USA; 2 Department of Radiology, Kings County Hospital Center, Brooklyn, USA

**Keywords:** ultrasound (u/s), computed tomography (ct) imaging, peritoneal implants, ruptured dermoid, ovarian teratomas

## Abstract

Ovarian neoplasms are categorized based on histopathologic features into epithelial surface cell tumors, germ cell tumors (teratomas), sex cord-stromal tumors, and metastases. Teratomas are the most common ovarian germ cell neoplasms. They are generally slow-growing lesions and can get fairly large before becoming symptomatic. The lesions are often incidentally discovered during imaging for other diagnostic purposes. Complications are uncommon but occur more commonly with larger lesions and include torsion, malignant degeneration, rupture, and rarely infection. When sizable, ovarian dermoid can rarely rupture and result in spillage of proteinaceous content into the peritoneal cavity, which can lead to chemical peritonitis. Additionally, the lesion can fragment into smaller lesions and can get implanted at different sites within the abdomen and pelvis. We present a case with an atypical presentation of a ruptured dermoid in a patient presenting with right upper quadrant pain who underwent sonographic evaluation, which demonstrated acute calculus cholecystitis but incidentally was found to have a partially calcified right subphrenic mass. Subsequent evaluation with computed tomography (CT) demonstrated multiple scattered peritoneal and mesenteric masses containing fat and calcification, highly suggestive of a chronically ruptured dermoid cyst.

## Introduction

Ovarian neoplasms are histopathologically categorized into epithelial surface cell tumors, germ cell tumors, sex cord-stromal tumors, and metastases. Teratomas are the most common ovarian germ cell tumors and arise from pluripotent primitive germ cells. Ovarian teratomas include mature cystic teratomas (dermoid cysts), immature teratomas, and monodermal teratomas (e.g., struma ovarii, carcinoid tumors, and neural tumors) [1]. Teratomas are most often seen in the ovaries but may also develop at other sites when germ cells become arrested during their migration from the allantois to the gonads [2,3]. Mature teratomas are more frequently seen in premenopausal females, with a mean patient age of 30 years [4]. The lesions are unilateral in up to 90% of cases and bilateral in approximately 10% [5]. The tumors generally have an indolent course and can grow fairly large before causing symptoms. They are often incidentally discovered on imaging. Complications of large lesions include torsion, malignant degeneration, and rupture and rarely infection. The spillage of contents from a ruptured cyst into the peritoneal cavity can cause aseptic inflammation and is termed "chemical peritonitis" [6]. The main risk factors for rupture include mechanical pressure from a gravid uterus, torsion with infarction, or direct trauma.

## Case presentation

**Figure 1:**
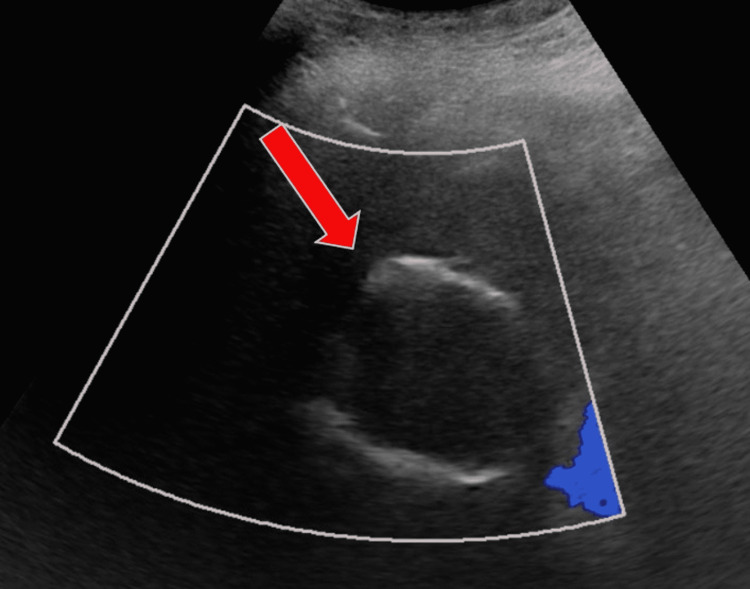
Ultrasound with color doppler along the hepatic dome demonstrates a peripherally echogenic round avascular mass (red arrow), which is centrally obscured by the echogenic walls.

**Figure 2:**
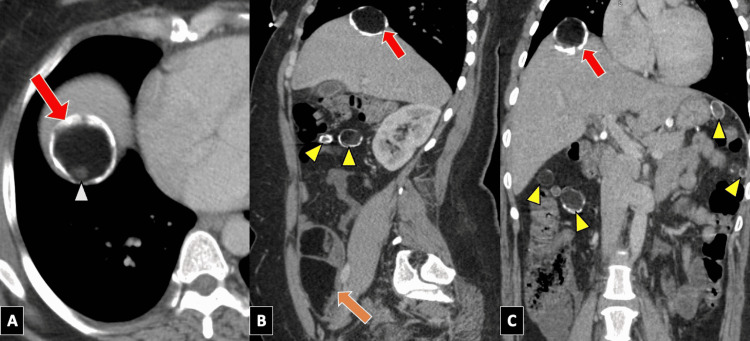
Axial (A), sagittal (B) and coronal (C) contrast-enhanced abdominal CT demonstrating a complex adnexal mass with fat and soft tissue attenuation (orange arrow). A partly calcified mixed attenuation mass is also seen at the dome of the liver (red arrows), representing one of many peritoneal deposits from a ruptured ovarian dermoid. This mass corresponds to the lesion seen on the initial ultrasound. A rounded soft tissue component within this lesion represents a characteristic Rokitansky nodule (white arrowhead). Multiple additional peritoneal and mesenteric dermoid implants are also seen (yellow arrowheads). CT: computed tomography.

A 46-year-old female with no pertinent medical history presented with a one-day history of right upper quadrant abdominal pain with associated nausea and non-bilious, non-bloody emesis. Physical examination was significant for right upper quadrant tenderness to palpation with concern for acute cholecystitis. The subsequent sonographic evaluation demonstrated an intraluminal echogenic shadowing structure within the gallbladder with gallbladder wall thickening and a positive sonographic Murphy's sign, suggestive of acute cholecystitis. Additionally, the sonogram demonstrated a heterogeneous, partially echogenic right subphrenic mass measuring approximately 2.9 cm along the hepatic dome with posterior acoustic shadowing (Figure 1). Subsequent computed tomography (CT) revealed a complex 15 cm adnexal mass with mixed soft tissue and fat attenuation with coarse scattered calcifications. Additionally, multiple smaller peripherally calcified masses (measuring up to 2.5 cm) with soft tissue and fat attenuation were noted throughout the peritoneum and interposed between the right hemidiaphragm and liver (Figure 2). Differentials included ruptured mature cystic teratoma with multiple intra-abdominal implants or an immature cystic teratoma with scattered metastatic implants. Pelvic ultrasound was not performed as the patient did not report pelvic pain, and CT findings were deemed sufficient. Laboratory tests revealed an elevated carbohydrate antigen (Ca 19-9) level. Cancer-antigen 125 (CA 125), alpha-fetoprotein (AFP), carcinoembryonic antigen (CEA), and beta-human chorionic gonadotropin (B-HCG) levels were normal. The patient was offered surgery for cholecystitis and removal of the peritoneal lesions for pathologic confirmation. However, the patient declined surgical intervention and was managed conservatively with antibiotics and analgesia. Repeat CT imaging eight weeks later did not demonstrate any significant interval change in the size and morphology of the peritoneal lesions, suggestive of stability.

## Discussion

Mature ovarian cystic teratomas or dermoid cysts are germ cell neoplasms that can contain tissues of any or all the germ cell layers (e.g., endodermal, ectodermal, and mesodermal origin), including but not limited to adipose tissue, calcifications or dental tissues, hair and even heterotopic tissues (e.g., thyroid tissue), which impart them with a varied but characteristic appearance on imaging [1]. 

Ultrasound is the preferred imaging modality owing to the lack of ionizing radiation and a dynamic mode of imaging with varying characteristics and can demonstrate a heterogeneous, complex cystic adnexal mass of varying echogenicity and echotexture depending on lesional composition. An echogenic interface at the edge of the lesion may obscure deeper structures (“the tip of the iceberg sign”) due to the composition of sebaceous or keratinaceous tissues (e.g., hair). Hair within the lesion may demonstrate thin linear echogenic bands (dot-dash pattern). Echogenic shadowing foci may represent lesional calcification or dental components, whereas non-shadowing echogenic foci may represent sebaceous or adipose tissues. Fluid-fluid levels may also be seen at the interfaces of different tissues, which can layer anti-dependently in discrete pockets in cases of rupture. Additionally, a characteristic eccentric echogenic mural nodule may also be demonstrated within the lesion representing a dermoid plug (Rokitansky nodule), which can contain a wide range of tissues including sebaceous, adipose, calcific, dental, keratinaceous (hair) components [1].

CT demonstrates high sensitivity for the diagnosis of cystic teratomas as well, however, is less utilized due to the risks of ionizing radiation. CT imaging characteristics classically show an adnexal/ovarian lesion containing regions of low or fat attenuation, which may contain fat-fluid levels, calcifications (maybe dentiform), and hair with or without the presence of a Rokitansky nodule [1].

MRI imaging may be utilized for troubleshooting in difficult cases. Characteristically, the utilization of fat-suppression sequences and chemical shift imaging will identify the presence of fat within the lesion, improving diagnostic yield. Lesional signal characteristics will vary based on tissue composition, with post-contrast enhancement adding diagnostic value to identify solid enhancing and potentially invasive/malignant components [1]. 

Complications, although rare, tend to occur in larger lesions (>6 cm) and include ovarian torsion, rupture, infection, and malignant transformation. The reported rate of a ruptured teratoma is less than 1% [4]. Rupture can be spontaneous or secondary to inciting events such as pelvic trauma, torsion, mass-effect from a gravid uterus, or surgical manipulation [7]. Clinically, it can present as acute chemical peritonitis secondary to peritoneal spillage of proteinaceous/sebaceous contents. An acutely ruptured ovarian teratoma can be diagnosed on CT when perilesional stranding or free fluid is seen. Subtle ruptures can lead to slow leakage of contents into the peritoneal cavity, resulting in an inflammatory response and can lead to the development of granulomas, which appear as nodular peritoneal implants, mimicking peritoneal carcinomatosis [8]. Delayed complications of chemical peritonitis can present as adhesional bowel obstruction, abdominal abscesses, and enterocutaneous fistulas that were not seen in our case [9]. 

Frank fragmentation of the primary lesion with scattered heterogenous fat-containing implants is highly suggestive of rupture, as in our case. Interestingly, the patient’s acute presentation was likely secondary to cholecystitis, and the incidental right subphrenic lesion likely represented sequela of a chronically ruptured dermoid cyst given the lack of pelvic pain and absence of characteristic imaging findings to suggest acute rupture, as previously described. Additionally, the patient did not report any prior typical symptomology or remote trauma to suggest prior dermoid rupture.

## Conclusions

Mature ovarian teratomas are relatively common benign lesions typically encountered in young premenopausal females. Complications are rare but typically occur in larger lesions (>6 cm) and include torsion, infection, rupture, and very rarely malignant degeneration. We summarized an interesting instance of a chronically ruptured dermoid cyst in a patient with right upper quadrant pain. Rupture is very rarely encountered, and fragmentation of the primary lesion with scattered peritoneal and mesenteric implants is a highly suggestive finding, as seen in our case.
